# Biomarkers for Gastric Cancer Screening and Early Diagnosis

**DOI:** 10.3390/biomedicines9101448

**Published:** 2021-10-12

**Authors:** Cristina Herrera-Pariente, Sheyla Montori, Joan Llach, Alex Bofill, Eduardo Albeniz, Leticia Moreira

**Affiliations:** 1 Centro de Investigación Biomédica en Red de Enfermedades Hepáticas y Digestivas (CIBEREHD), Gastroenterology Department, Institut d’Investigacions Biomèdiques August Pi i Sunyer (IDIBAPS), University of Barcelona, 08036 Barcelona, Spain; cristina.herrera@ciberehd.org (C.H.-P.); jllachr@clinic.cat (J.L.); BOFILL@clinic.cat (A.B.); 2 UPNA, IdiSNA, Navarrabiomed Biomedical Research Center, Gastrointestinal Endoscopy Research Unit, 31008 Pamplona, Spain; sheyla.montori.pina@navarra.es (S.M.); edualbeniz@hotmail.com (E.A.); 3 Endoscopy Unit, Gastroenterology Department, Complejo Hospitalario de Navarra, 31008 Pamplona, Spain

**Keywords:** gastric cancer, diagnostic, noninvasive biomarker, liquid biopsy, screening

## Abstract

Gastric cancer is one of the most common cancers worldwide, with a bad prognosis associated with late-stage diagnosis, significantly decreasing the overall survival. This highlights the importance of early detection to improve the clinical course of these patients. Although screening programs, based on endoscopic or radiologic approaches, have been useful in countries with high incidence, they are not cost-effective in low-incidence populations as a massive screening strategy. Additionally, current biomarkers used in daily routine are not specific and sensitive enough, and most of them are obtained invasively. Thus, it is imperative to discover new noninvasive biomarkers able to diagnose early-stage gastric cancer. In this context, liquid biopsy is a promising strategy. In this review, we briefly discuss some of the potential biomarkers for gastric cancer screening and diagnosis identified in blood, saliva, urine, stool, and gastric juice.

## 1. Introduction

Gastric cancer (GC) is one of the most common malignancies worldwide. It ranks fifth in terms of incidence and third in terms of mortality, with more than 1,000,000 new cases and nearly 800,000 deaths in 2018 [1]. The major risk factors are age, obesity, smoking, alcohol, family history, and *Helicobacter pylori* infection [2]. The latter is one of the most important; thus, it was classified as a class I carcinogen by the Agency for Research on Cancer. The most important environmental factor for the intestinal GC subtype is a persistent infection of *Helicobacter pylori*, since it leads to chronic inflammation and to the appearance of other precursor lesions of GC: atrophic gastritis, intestinal metaplasia (IM), and dysplasia (known as Correa’s cascade). Hence, identification of *Helicobacter pylori* infection with classical culture, as well as novel molecular methods, is very useful to increase the knowledge about this bacterium and to improve its diagnosis and eradication [3].

There is a high similarity between incidence and mortality rates, reflecting the bad prognosis of this cancer. The reason is that GC patients are usually asymptomatic at the beginning of the disease and, hence, diagnosis is frequently performed in advanced stages [4]. As a consequence, the 5 year overall survival of patients diagnosed in advanced stages is less than 5% due to the tumor already spreading to distant organs [5]. Therefore, early detection is necessary to improve the clinical course of GC patients.

Currently, the gold standard method to detect GC is upper endoscopy in combination with tissue biopsy. This method has sensitivity and specificity values of 69% and 96%, respectively [6]. However, it is an invasive, costly, and time-consuming methodology. In countries where there is a high incidence of the disease, screening programs have been performed with the aim of reducing morbidity and mortality [7]. These programs have been useful in order to detect early GC, increasing the number of treatable cancers and improving the 5 year overall survival [8]. However, they are based on endoscopic or radiologic approaches, and, in countries with low incidence, such as Western countries, it would not be cost-effective as a massive screening strategy [9,10]. Accordingly, the implementation of other methodologies, such as biomarkers, is mandatory to enhance GC early diagnosis and the overall survival of the patients. Currently, the biomarkers most frequently used in clinical routine to detect GC are carcinoembryonic antigen (CEA) or carbohydrate antigens such as CA19-9 or CA72-4. However, their sensitivity and specificity are limited, they are not unique for GC diagnosis [11], their positive rate to detect early GC is very low [12], and they are not useful in the detection of premalignant GC lesions such as atrophic gastritis, IM, and dysplasia. Thus, the discovery of new biomarkers is still necessary to diagnose GC at these earlier stages to increase the overall survival.

Biomarkers are biological molecules that are involved entirely or partially in carcinogenesis processes and, accordingly, can highlight abnormal changes in the patient [13]. Ideally, they should be noninvasive, sensitive, specific, cost-effective, and easy to implement in clinical routine. Taking this into account, liquid biopsy represents a good approach to achieve this objective. In this context, blood, urine, stool, saliva, or gastric juice could be interesting fluids to find new noninvasive biomarkers. In this review, a brief updated overview of potential noninvasive biomarkers for GC screening and early diagnosis is discussed. 

To that end, relevant and original studies published in English were collected from PubMed from 2000 to 2021, using the following keywords: “biomarker” or “screening biomarker” and “gastric cancer” or “stomach cancer” or “early gastric cancer”.

## 2. Blood Biomarkers

A great variety of different molecules in blood have been associated with GC carcinogenesis. Among them, DNA, proteins, and noncoding RNA molecules such as microRNAs (miRNA), circular RNAs (circRNA), and long noncoding RNAs (lncRNA) can be highlighted. These molecules enable the reliable discovery of new noninvasive biomarkers for GC in blood. Blood samples have several advantages over the endoscopy or tissue biopsy, especially because they are minimally invasive, easy to obtain, and very common in clinical routine. In addition, it is quite accepted by the general population and its cost is sustainable.

### 2.1. Circulating Free DNA

When a tumor grows and develops, circulating free DNA (cfDNA) is released into the blood, facilitating its detection. This makes cfDNA a promising strategy for early diagnosis [14]. Therefore, some studies have evaluated its implication as a useful biomarker. In the last years, two studies [15,16] identified that cfDNA was able to distinguish GC patients from healthy individuals with an area under the curve (AUC) of 0.94 and 0.99, respectively. Nevertheless, the use of cfDNA for early GC detection is difficult to implement due to its low abundance in the early stage and the technical challenges in its detection [17]. Another approach was evaluated by Cohen et al., where the authors designed the CancerSEEK blood test. It is based on the evaluation of mutations in cfDNA in combination with circulating proteins. Using this strategy, they were able to detect patients with eight common cancer types, including GC, with a sensitivity value around 70% [18]. However, prospective studies need to be performed.

Several studies have identified DNA hypermethylation as a common epigenetic event in GC carcinogenesis. However, DNA methylation can occur in noncancerous cells, and biomarkers must be specific [19]. Accordingly, *RUNX3*, *SFRP2*, or *RPRML* are some of the methylated genes suggested as noninvasive biomarkers for early detection of GC [20,21,22]. Among them, *SFRP2* obtained a sensitivity of 60.9% and specificity of 86.0% in a cohort of 92 GC patients and 50 controls. Moreover, this biomarker was also able to identify IM patients with a sensitivity of 56.3%. However, the differences between the positive detection rates of plasma methylated *SFRP2* in GC and IM patients was not significant, probably because the number of patients with IM was very low [21]. Although further studies are needed, it could be an interesting biomarker for screening GC and premalignant GC lesions.

In this context, identification of premalignant gastric lesions would help to diagnose GC earlier. Some GCs, particularly intestinal GC, develop in a continuous process from non-atrophic gastritis, leading to chronic atrophic gastritis (CAG) to IM and dysplasia, and finally to intestinal GC. Thus, identifying biomarkers able to detect these steps will be helpful. Better results than *SFRP2* have been found with *RPRML*, also known as Reprimo-like. It has been found methylated not only in GC patients, but also in IM patients, finding higher levels in GC, then in dysplasia, and lower in IM. In this study, the authors analyzed a total of 150 plasma samples (50 GC patients, 50 dysplasia, 50 CAG patients with IM, and 30 healthy individuals). As a conclusion, the authors suggested that methylation of the promoter region of *RPRML* gene might be an early event during gastric carcinogenesis [23]; thus, it could be an interesting biomarker for early GC.

Mitochondrial DNA has also been proposed as a biomarker in blood because higher levels have been seen in GC patients in comparison with healthy individuals. The authors identified three intervals (I, II and III) of increasing mtDNA values (<2, 2–20, and >20). Interval I was composed of non-atrophic gastritis and a few GC samples, interval II was composed of healthy controls, and interval III was composed exclusively of GC patients. The authors suggested that patients with low mtDNA should be periodically monitored by endoscopy because they are likely to present precancerous or GC lesions. However, some patients with the same mtDNA levels as the asymptomatic group presented precancerous and GC lesions; hence, further studies will be needed in order to clarify this fact. In this context, the authors suggested that mtDNA level may be combined with IL-8 levels to increase its diagnostic value, although this should be further validated [24].

### 2.2. Noncoding RNA

#### 2.2.1. miRNA

miRNAs are short noncoding RNAs of 20–25 nucleotides. They play a crucial role in various cellular functions, such as differentiation, growth, and apoptosis, and they are involved in gastric carcinogenesis [25]. In addition, their identification can be performed by a variety of routine laboratory techniques such as quantitative reverse transcription polymerase chain reaction (qRT-PCR) or microarrays. Therefore, biomarkers based on miRNA expression seem to be feasible for GC diagnosis. In this context, different strategies have been followed.

Recently, Yao et al. performed differential expression analysis of miRNA profiles using two datasets from plasma samples, discovering three miRNAs (miR-320a, miR-1260b, and miR-6515-5p) with an AUC higher than 0.95 in all cases. They validated them in two additional external datasets obtaining an AUC higher than 0.946 in all the miRNAs. In the whole study, 235 GC patients and 3174 healthy controls were analyzed, reflecting a real-life scenario, where the proportion of GC among healthy individuals is low. However, despite these promising data, it would be interesting to further evaluate these miRNAs in early GC patients, GC premalignant lesions, and other types of cancer to determine if they can be found at these less advanced stages and if they are exclusive for GC or not [26].

Another interesting strategy is the use of extracellular vesicles. Among them, exosomes have shown an interesting role in identifying new diagnostic biomarkers, although they are involved in other functions such as immune response, metastasis, or drug resistance [27]. Exosomes are extracellular vesicles about 100 nm in diameter that contain DNA, RNA, or proteins and are released from cells. In addition, they prevent miRNA degradation by RNase [28]; hence, several studies have been focused on exosomal miRNAs. In this context, Zheng et al. studied exosomal miR-590-5p by qRT-PCR in 168 GC and 50 control serum samples. The expression of exosomal miR-590-5p was found to be lower in GC patients than in the healthy control group. The levels were also lower in the late-stage GC when compared with the early stages, suggesting that this exosomal miRNA could be a promising biomarker for early GC detection. This study also compared this biomarker with traditional serum ones such as CA72-4, CEA, and CA19-9, obtaining the best sensitivity and accuracy values for GC diagnosis using exosomal miR-590-5p. The AUC for this miRNA was 0.81, with a sensitivity of 63.7% and specificity of 86.0%, and, although validation in a higher external cohort is necessary, this is an interesting strategy for further analysis [29].

Exosomal miR-1246 has been found upregulated in serum samples of GC patients by qRT-PCR. It was able to discriminate them from healthy individuals with an AUC of 0.91, with sensitivity and specificity values of 82.3% and 86.0%, respectively. A total of 85 GC patients, 30 patients with benign gastric diseases (gastric ulcer, atrophic gastritis and gastric polyps), and 50 healthy individuals were analyzed. The authors also found that circulating exosomal miR-1246 levels gradually increased from stage I–II patients to stage III–IV patients. Furthermore, this miRNA was able to distinguish stage I patients from healthy individuals and from benign gastric disease patients with an AUC of 0.84 and 0.81, respectively [30]. This makes miR-1246 an interesting biomarker for early GC diagnosis.

Moving on from exosomes, other miRNAs such as miR-376C, miR-21 or miR-101-3p have shown interesting results [31,32,33]. Among them, serum miR-101-3p can be highlighted. In a study, miR-101-3p was measured in 61 atrophic gastritis patients, 86 GC patients, and 50 healthy controls by qRT-PCR. The authors observed that miR-101-3p exhibited a high diagnostic value on atrophic gastritis and GC, with an AUC of 0.85 and 0.87, respectively. However, to distinguish atrophic gastritis patients from GC patients, the best approach was the combination of miR-101-3p with pepsinogen I and pepsinogen I/II (PGI:II) ratio, with an AUC of 0.856 [33].

As can be seen, the combination of different biomarkers can improve the diagnostic performance. Given this, different combinations have been proposed. For example, a combination model of age and two miRNAs (miR-19-3p and miR483-5p) called the Pre-Cancer Screening Model was developed. The cohort included 70 gastritis, 20 low-grade intraepithelial neoplasia, 34 high-grade intraepithelial neoplasia, and 56 early GC subjects. This model showed an AUC of 0.84 with sensitivity and specificity values of 87.7% and 62.8%, respectively, to diagnose early GC and precancerous lesions, showing better performance than any single miRNA. However, multicenter validation is still needed before using Pre-Cancer Screening Model in clinical routine [34].

Zhu et al. analyzed plasma of 128 gastric non-cardia adenocarcinoma and 182 controls using TLDA chips and qRT-PCR. After discovery, training, and validation phases, they concluded that a five-miRNA panel (miR-16, miR-25, miR-92a, miR-451, and miR-486-5p) was the best strategy to distinguish gastric non-cardia adenocarcinoma from healthy individuals with an AUC of 0.812, sensitivity of 72.9%, and specificity of 89.2%. They also performed an external validation phase using qRT-PCR with 36 gastric cardia adenocarcinoma and 36 gastric non-cardia adenocarcinoma patients, in different stages (Ia/Ib and III/IV), to further asses their functionality in all GC patients. They found that these five miRNAs were overexpressed in all GC groups, suggesting that they may be useful for the early diagnosis of GC patients. This study reinforces the idea that circulating miRNAs are novel, noninvasive diagnostic biomarkers with good results for mass screening or early GC detection [35].

Another miRNA panel, in this case using serum samples, was recently described using a multitarget miRNA assay in more than 5000 individuals from Singapore and Korea. This panel was based on 12 miRNAs, obtaining, in the prospective phase, an AUC of 0.848 and sensitivity and specificity values of 87% and 68.4%, respectively, to distinguish GC from healthy controls. They showed that, using their miRNA panel, they were able to reduce the number of endoscopies from 40 to 15 to detect one GC patient in this population and to increase the compliance as a mass screening strategy, as it is a noninvasive test compared with upper endoscopy. As previously seen in other studies, the authors confirmed that combination of this 12-miRNA panel with patient age, *Helicobacter pylori* serology, and PGI:II ratio improved the AUC to 0.884 and specificity to 69.4%, while maintaining the sensitivity value, for GC detection. This reinforces the idea that the combination of different types of biomarkers increases the detection accuracy [36].

#### 2.2.2. circRNA

circRNA is also a noncoding RNA that is generated from introns or exons through loop introns or reverse splicing. It has shown advantages as a biomarker for tumor diagnosis because of its biological properties and functions. Thus, some circRNA candidates have been evaluated. For example, circ_0000520 levels were detected using qRT-PCR in plasma samples in a cohort of 62 patients (17 healthy and 45 GC patients). They found that it was downregulated, and it was able to distinguish GC among healthy individuals with an AUC of 0.8967, sensitivity of 82.35%, and specificity of 84.44% [37]. However, the cohort was small; thus, more samples need to be analyzed in further studies. 

As previously seen with miRNAs, exosomal circRNAs have also been studied. Shao et al. identified, by qRT-PCR, that circ_0065149 levels in plasma exosomes from early GC patients were significantly lower than those from healthy individuals (*p* < 0.001). Thus, circ_0065149 can distinguish early GC patients from healthy patients with an AUC of 0.64, sensitivity of 48.7%, and specificity of 90.2%, using a cohort of 80 individuals (41 healthy controls and 39 early GC patients). Interestingly, the authors analyzed this circRNA in gastric juice, although no differences were found among gastric ulcer, chronic atrophic gastritis, GC, and healthy patients [38].

As mentioned above, the combination of different biomarkers increases detection ability, making up the limitations of a single marker, and this can also be applied to circRNAs. For example, plasma circ_0000745 increased the AUC from 0.683 to 0.775 when combined with CEA level to distinguish GC patients from healthy individuals. However, sensitivity decreased while specificity increased from 45% to 63.3% [39]. Similar results were obtained when combining plasma circPTPN22 with CEA and CA-199 levels. In the latter case, AUC, sensitivity, and specificity levels were 0.892, 83%, and 87%, respectively. The authors also realized that plasma levels of circPTPN22 increased gradually from healthy donors to GC patients, passing through patients with gastritis, making circPTPN22A an interesting approach for clinical routine [40].

#### 2.2.3. lncRNA

lncRNAs are long (>200 nucleotides) transcripts with null or restricted ability to encode proteins. They regulate various biological processes, are highly stable, and circulate in body fluids, and some studies have analyzed their diagnostic role.

In this context, Dong et al., identified a three-lncRNA signature (CUDR, LSINCT-5, and PTENP1) as a potential diagnostic marker to distinguish GC from healthy individuals with an AUC of 0.829. Additionally, they also analyzed the levels of this lncRNA signature in an external cohort (18 healthy individuals, 15 gastric peptic ulcer subjects, nine early-stage GC, and 21 later-stage GC) and concluded that this signature was able to distinguish healthy samples from early-stage (stage I) and later-stage (stage III–IV) GC with an AUC of 0.832 and 0.828, sensitivity of 77.8% and 85.7%, and specificity of 97% and 84.8%, respectively. This study reinforces the role of these three serum biomarkers for GC diagnosis, especially for early-stage GC [41].

Following a similar strategy, Zhang et al. identified five upregulated plasma lncRNAs (TINCR, CCAT2, AOC4P, BANCR, and LINC00857) that were able to distinguish GC patients with an AUC of 0.90 in the validation cohort (80 GC patients and 80 healthy individuals). Interestingly, they also evaluated if this lncRNA signature was able to distinguish patients with similar symptoms but different diseases (precancerous lesions or gastrointestinal stromal tumors). They found that this lncRNA signature was able to distinguish GC from precancerous patients with a sensitivity of 68% and specificity of 89% and to distinguish GC from gastrointestinal stromal tumors with a sensitivity and specificity of 68% and 86%, respectively [42].

Another lncRNA that has been identified in GC patients is LINC00152. A study included plasma from 79 GC patients, 31 gastric epithelial dysplasia patients, and 81 healthy volunteers. They found that LINC00152 level was elevated in plasma samples of GC patients when compared with healthy individuals (*p* < 0.001), although no significant differences were found between patients with gastric epithelial dysplasia and healthy controls. Additionally, they found that this lncRNA is also contained in exosomes, suggesting that these exosomes are protecting plasma lncRNAs [43].

Another lncRNA that has also been found in exosomes is lncUEGC1. Using plasma samples from 10 stage I GC patients and five healthy individuals, 79 upregulated exosomal lncRNAs were identified. These results were combined with RNA sequencing from GC cell lines, and then validated by qRT-PCR in 51 stage I–II GC patients and 60 healthy controls. In the end, they focused on lncUEGC1 and lncUEGC2. The best results were obtained using lncUEGC1 being able to distinguish stage I–II GC patients from healthy controls with an AUC of 0.88, while lncUEGC2 and serum CEA showed lower AUC values (0.76 and 0.66, respectively). Exosomal lncUEGC1 was also able to distinguish stage I GC patients (*n* = 23) from chronic atrophic gastritis (*n* = 18) with an AUC of 0.84. This highlights how lncUEGC1 may be promising for early GC biomarker development. Additionally, it is the first article focusing on long-chain RNA sequencing that thoroughly screened potential early GC biomarkers [44].

In a similar context, serum exosomal lncRNA HOTTIP was found upregulated in GC patients compared to normal controls. They analyzed 126 GC patients and 120 healthy people by qRT-PCR. The AUC was 0.83, and sensitivity and specificity were 69.8% and 85.0%, respectively. These results were better than those obtained with CEA, CA 19-9, and CA72-4 or even their combination. The best result (AUC of 0.87) was obtained combining exosomal lncRNA HOTTIP with CEA, CA 19-9, and CA72-4, which supports the idea that the combination of different biomarkers can improve GC detection [45]. Further analysis focusing only on early GC patients will be useful to make it more feasible to be used as a GC screening biomarker in clinical routine.

To sum up, RNA molecules are promising biomarkers for GC screening. Extensive studies have been performed, and several molecules seem to be interesting biomarkers. However, further validation in larger cohorts is still necessary in order to confirm their utility in clinical practice.

### 2.3. Proteins

As seen previously with the CancerSEEK test, proteins are also interesting molecules for GC screening. Among them, the most interesting approach could be a protein signature or panel, although some studies focused on only one protein [46]. Performing ELISA on plasma samples from 37 GC patients and 34 healthy controls, Song et al. evaluated a four-protein signature (ATP5B–ATP5O–NDUFB4–NDUFB8) obtaining an AUC of 0.778. It was lower that the AUC found in tissue biopsy for the same protein signature, probably because of differences between experimental tests [47]. Better results were obtained by Tong et al., identifying another protein signature (VEGF, ADAM8, IgG for *Helicobacter pylori*, serum pepsinogen I, and pepsinogen II) with AUC, sensitivity, and specificity values of 0.85, 88.6%, and 83.2%, respectively [48].

Another interesting approach is to combine new biomarkers with previous biomarkers already used in clinical routine. Accordingly, Liu et al. identified that the combination of SLC6A3, CEA, and CA19-9 permitted distinguishing GC from healthy volunteers with an AUC of 0.818, higher than that obtained with SLC6A3 alone. However, the specificity decreased [49].

On the other hand, protein profiles of early GC were significantly different from those of advanced stage GC, and biomarkers found in advanced GC have limited reference value for early GC screening. Therefore, another recent study used plasma samples from 15 early GC patients and 15 healthy controls. From the total of 2040 proteins identified, 11 proteins were found to be differentially expressed. The best result was achieved with a combination of 11 proteins, obtaining AUC, sensitivity, and specificity values of 0.711, 66.7%, and 86.7%, respectively. This model showed that the changed proteins identified by plasma proteomics could help distinguish early GC patients from healthy controls. However, the authors believe that this method has the ability to develop a more accurate diagnosis tool for early GC with a larger sample size [50].

As previously mentioned, focusing on the detection of premalignant lesions is a good strategy to improve GC diagnosis; hence, several proteins have been proposed. 

Analyzing the serum from 72 patients by ELISA, the authors found that the best way to identify corpus CAG/IM was using the serum PGI:II ratio. The AUC value was 0.902, with sensitivity and specificity values of 83.3% and 77.9%, respectively. Worse results were obtained using only PGI, with an AUC of 0.659, sensitivity of 66.7%, and specificity of 85.3%. In addition, poor test performances were found using serum PGII levels [51]. In this study, the authors mentioned that, using the manufacturer-recommended cutoff value, sensitivity decreased while specificity increased. Therefore, the authors suggested increasing the cutoff value for the ratio. However, in a prospective study using CAG Spanish patients, the authors found that the PGI, PGII, and PGI:II ratio showed no significant differences; hence, these biomarkers were not good enough in this scenario [52]. Accordingly, larger cohort studies will be needed to determine the real value for the diagnosis of CAG/IM before its application in clinical practice.

Other interesting protein is serum trefoil factor 3 (TFF3). This protein has been found increased in serum from GC patients with AUC of 0.89, with sensitivity of 80.9%, and specificity of 81.0% [53]. In this context, another study found that early GC showed higher levels of TFF3 using ELISA in plasma samples, although no differences were found between CAG and normal controls. For stage I GC patients, AUC was 0.703 and sensitivity and specificity were 83.3% and 54.5%, respectively [54]. Another study compared the PGI:II ratio with TFF3 in serum samples in CAG patients, showing that the PGI:II ratio AUC value was better (0.76 and 0.53, respectively) [55].

Another protein biomarker that could be useful to identify premalignant lesions is high-sensitivity C-reactive protein (hs-CRP). Altun et al. studied serum levels of 811 patients, finding that those patients with chronic gastritis had higher levels of hs-CRP, although the authors suggested that this biomarker is not useful for cancer or precancerous lesions screening [56]. In another study on 378 patients, the authors found that a combination of serum hs-CRP levels and PGI:II ratio showed sensitivity and specificity values of 67% and 72% for the detection of IM patients. In addition, hs-CRP was good enough to detect locally advanced GC (sensitivity of 67% and specificity of 85%) [57].

Another promising protein is macrophage migration inhibitory factor (MIF), which was detected by ELISA in higher levels in serum patients with gastric inflammation and even higher in IM and GC patients. In the cohort, there were 15 healthy, 15 chronic antral gastritis, 20 IM, and 40 GC patients. It is important to highlight that all chronic gastritis patients were *Helicobacter pylori*-positive, while not all IM and GC patients were positive; thus, MIF levels in IM and GC were independent of *Helicobacter pylori* infection. This suggests that MIF is an interesting biomarker for the early detection of GC [58]. However, studies in larger external cohorts are still needed.

Regarding *Helicobacter pylori* infection, it has been found that the seropositivity of specific antigens is associated with the risk of gastric lesions. Anti-CagA antibodies have been related to an increased risk of CAG, IM, and dysplasia. Additionally, anti-VacA and anti-GroEL have been related only to an increased risk of CAG. In addition, for anti-CagA and anti-VagA antigens, an increased risk of progression was found [59]. This interesting finding should be borne in mind for further studies, since a high percentage of the population has had a *Helicobacter pylori* infection during their life.

As outlined above, the use of blood biomarkers has been extensively studied because blood is very easy to obtain routinely in hospital laboratories and is widely accepted by patients. However, the use of some of these biomarkers still has limitations. Thus, the use of cfDNA for early GC detection is difficult to implement due to the low abundance in early-stage GC and the technical challenges in its detection [17]. Furthermore, cfDNA is derived from cells that undergo necrosis or apoptosis, which might not reflect viable tumor cells [60]. In this regard, the use of blood exosomes containing nucleic acids and proteins is in the spotlight, since they are vesicles actively released from viable tumor cells and, therefore, could better reflect tumor dynamics [61]. Moreover, exosomes increase biomarker stability as they protect them from degradation [62,63,64,65]. Nonetheless, the use of exosomes to isolate biomarkers also faces several issues. First, exosomes must be isolated quickly and accurately, and the techniques are cumbersome, time-consuming, and costly. Secondly, the low yield and low purity limit its clinical application. Moreover, it is necessary to distinguish the source of exosomes in the blood to know if they are released by normal cells or by tumor cells [66,67,68]. Therefore, the standardization of protocols and identification methods is necessary to use exosomes widely in clinical practice. On the other hand, the lack of specificity of some of these biomarkers [19] requires their combination in order to be applied clinically. In this sense, biomarker panels are already a reality; however, as the profiles of expression may change during the development of tumors [50], it would be interesting to design a panel that encompasses biomarkers at all stages in order to evaluate the stage of development of the lesion without the need for a solid biopsy. Lastly, it would perhaps be more useful to validate documented biomarkers on a larger scale to create new panels that can be used in multiple fields [68]. Therefore, more external, multicenter validation studies that include patients from different populations and with different stages of the lesion are needed to evaluate the biomarkers that can be detected at earlier stages to be able to treat the GC in time.

Table 1 summarizes the potential biomarkers in blood (serum or plasma) for GC screening and early diagnosis.

## 3. Saliva Biomarkers

The use of saliva as a source for biomarkers is another interesting approach for GC screening since its collection is minimally invasive and salivary glands are stimulated by mediators that can be released from tumors. In this context, different biomarkers have been identified. A panel that combines three mRNAs (SPINK7, PPL, and SEMA4B), two miRNAs (miR140-5p and miR301a), and demographic data (smoking, gender, and age) was discovered and validated in a cohort of 163 GC patients and 131 healthy volunteers. They obtained an AUC in the validation set of 0.87 and sensitivity and specificity values of 82% and 77%. However, this study was performed in a high-risk cohort (Korean population); therefore, further studies in the Western population will be needed to confirm if this panel is also helpful in clinical routine in low-prevalence countries [69].

Another panel that combines salivary mRNA PPL (using qRT-PCR) with CEA level in blood samples achieved a sensitivity of 92% and specificity of 87%, in a cohort of 200 GC patients and 200 healthy volunteers [70]. This study highlights the possibility of improving the diagnostic ability by mixing not only multiple biomarkers but also different samples. Thus, this strategy can be interesting to bear in mind for ameliorate screening strategies.

Two different studies also proposed salivary proteins as GC biomarkers. A protein panel composed of three proteins was obtained using quantitative proteomics and an ELISA approach in a cohort of 40 GC and 40 healthy individuals. It was able to distinguish GC patients with an AUC of 0.93. However, the cohort was small, and replication in a huge cohort is still needed [71]. A salivary glycopattern was also proposed by Shu et al. as a diagnostic biomarker for GC. Shu et al. studied a cohort of 87 GC patients, 54 patients with atrophic gastritis, and 60 healthy controls. They established two different models to distinguish atrophic gastritis and GC patients from healthy individuals, obtaining an AUC of 0.83 and 0.89, sensitivity of 92% and 96%, and specificity of 72% and 80% [72].

Saliva offers many biochemical advantages over blood and tissue, as it is noninvasive, easy to store, and cost-effective to collect, also in addition to causing less discomfort for the patient [73]. Molecular diagnosis based on saliva reflects the physiological conditions of the body and, therefore, it enables controlling not only oral but also systemic diseases [74]. Ongoing advances in saliva research have led to the scientific community coining the term “salivaomics” [75]. Saliva is a complex body fluid that contains a wide collection of proteins, as well as DNA, mRNA, miRNAs, metabolites, and microbiota [76], all of which can be used as biomarkers. Hence, one of its limitations is purification of the sample, which requires the application of different protocols depending on the target biomarker, while avoiding contamination with other molecules in the sample. Furthermore, saliva-based diagnosis in cancer is still in its infancy. In fact, most studies have not performed a correlation analysis between the salivary biomarkers and TNM stage [77]. In this regard, future guidelines on saliva research should require a randomized independent validation phase and multicenter design. Fortunately, ongoing advances in salivaomics technology point toward a promising future of salivary biomarkers in GC management among others.

Table 2 summarizes the potential biomarkers in saliva for GC screening.

## 4. Urine Biomarkers

Urine is another interesting biological fluid for the identification of new GC noninvasive biomarkers. Compared with serum, it is obtained more easily, in large amounts, and without invasion. Consequently, some biomarkers have been identified.

Iwasaki et al. identified that a miRNA panel (miR-6807-5p and miR-6856-5p) in combination with *Helicobacter pylori* status was able to distinguish GC patients from healthy individuals. The AUC was 0.885, with sensitivity and specificity values of 76.9% and 88.9%, respectively. This miRNA panel also permits distinguishing between healthy individuals and stage I GC with an AUC of 0.748, which is a notable benefit [78].

Proteins have also shown an interesting role as urine biomarkers, such as ADAM12 and MMP-9/NGAL [79]. In this context, interesting results were obtained using high-throughput quantitative proteomics. In this study, the authors identified two different panels, depending on gender, using a study population of 138 healthy patients 144 GC patients. For males, uTFF1, uADAM12, and *Helicobacter pylori* status were able to distinguish GC from healthy individuals with an AUC of 0.858, while, for females, using uTFF1, uBARD1, and *Helicobacter pylori* status, they obtained an AUC of 0.893. This panel also allowed identifying early GC with an AUC of around 0.850 for both sexes [80].

Lastly, 8-OHdG and 8-OHG, two oxidative nucleic acid modifications in DNA and RNA, respectively, have been detected in high urine levels in GC patients. Thus, 8-OHdG and 8-OHG could distinguish GC patients from healthy volunteers with an AUC of 0.777 and 0.841, respectively [81].

Urine is the carrier of blood wastes, being rich in intermediate or end-products of many metabolic pathways [82]. Therefore, urine may provide information from distant organs [83,84]. Nevertheless, these biomarkers can be shared by different types of cancer, making their origin difficult to ascertain. Thus, whether these biomarkers are specific to GC is a challenging issue [85]. This is one of the major limitations of using urine biomarkers in nonurological cancers. In this sense, the results achieved until now need to be validated with appropriate control groups, including other diseases or disorders with clinical and metabolic profiles close to those of the GC. On the other hand, many components may not get filtered in the urine compared to blood. Moreover, biomarker concentrations may change with hydration status, urine volume, medications, renal pathology, and other benign comorbidities [80,86]. Hence, it is very likely that the concentrations of biomarkers are not reliable, with a high degree of variability. Therefore, the experimental protocols that are adopted must be standardized. However, the lack of multicenter studies for the prospective validation of large cohorts remains the main barrier to its adoption in clinical practice.

Table 3 shows the potential urine biomarkers for GC diagnosis.

## 5. Stool Biomarkers

Similar to urine, obtaining stool does not require any type of invasion and, consequently, it has become a very useful sample for screening other types of cancer such as colorectal cancer. However, in GC, few studies have been performed. In 2003, fecal GP87 was proposed as a GC biomarker, obtaining a sensitivity of 79.3% and 84.4% for GC and chronic atrophic gastritis patients, respectively [87]. The authors suggested that this protein may help not only to diagnose GC, but also to select a subpopulation at high risk for GC that should be followed endoscopically. In the same year, M2-PK was proponed as a biomarker for GC, but low sensitivity was found [88]. However, although further studies should have been performed, no more information is currently available.

Some limitations have led to the fact that feces is not used frequently to detect GC biomarkers. Firstly, patients may be reluctant to handle fecal material. In addition, handling stool samples is challenging for both patients and investigators, and the quality of the sample collection by patients may affect the results [89]. Furthermore, as in the case of urine and blood, the biomarkers found in the stool may not be specific for GC. Nevertheless, recent studies have suggested a correlation between intestinal microbiota and the development of GC [90,91], which opens a new scenario for noninvasive biomarkers. 

Table 4 summarizes the most interesting stool biomarkers for GC screening.

## 6. Gastric Juice

Although gastric juice has been considered a waste product during gastroscopic examination, it can be another interesting biomarker source for GC screening and diagnosis, since it represents an exclusive stomach fluid. As it is obtained directly from the area of the lesion, dilution of the biomarkers and their lack of specificity are avoided [92]. Furthermore, gastric juice is secreted directly by the cells without being eliminated by the liver [93,94], which guarantees the adequate representation of all the molecules surrounding the lesion. Moreover, gastric juice can be easily obtained during diagnostic endoscopy in symptomatic patients or during surveillance of premalignant lesions. Despite all these advantages, this fluid has not been widely used to date due to certain limitations. First, it requires a specialist to obtain the sample. Nevertheless, although it is commonly obtained in an invasive way, if performed during the course of a clinically indicated gastroscopy, it does not increase the invasiveness of the procedure. Another limitation is the acceptance of the patients as a routine exam because gastroscopy often causes discomfort. However, there are other strategies such as endogastric capsule [95,96]. This makes feasible the collection of gastric juice in a minimally invasive way [97], and it is quite accepted by the patients [98]. Lastly, the use of gastric juice as a DNA biomarker is not feasible, since DNA is easily degraded due to gastric acidity. Alternatively, gastric lavages represent a potential method for the detection of DNA alterations [60].

Among the biomarkers identified in gastric juice, the majority are noncoding RNAs and some proteins, probably because, as previously said, DNA is easily degraded due to its gastric juice acidity.

### 6.1. DNA

To avoid DNA degradation by gastric acidity, exosomes have been proposed as an interesting solution. In this context, Yamamoto et al. identified that *BARHL2* methylation can discriminate GC patients from healthy individuals, obtaining an AUC of 0.923 with 90% and 100% sensitivity and specificity, respectively. They analyzed 10 early and 10 advanced GC patients of intestinal and diffuse types. Therefore, *BARHL2* methylation was found to be a good biomarker for GC detection, despite the small size of the cohort. In addition, this biomarker is not influenced by atrophic gastritis or *Helicobacter pylori* infection [99]. More studies in bigger cohorts should be performed to further validate *BARHL2* methylation as GC screening biomarker.

### 6.2. Noncoding RNA

#### 6.2.1. miRNA

Focusing on miRNAs, a cohort of 47 healthy patients, 42 GC patients, 34 gastric ulcer patients, and 18 atrophic gastritis patients was used to determine the expression of different miRNAs. In different studies, the authors focused on miR-129-1-3p and miR-129-2-3p [100], miR-421 [101], and miR-21 and miR-106a [102]. Among them, the most promising were miR-21 and miR-106a, being able to distinguish GC patients from healthy controls with an AUC of 0.969 and 0.871, respectively. They also evaluated the use of these biomarkers to identify early GC patients, since this is the most efficient approach to perform an early diagnosis. They found that a combination of both miRNAs with serum CEA was able to obtain a higher positive detection rate of early GC. However, it is well known that both miRNAs are involved in proliferation and oncogenic activity, and the authors found them in low levels in GC patients. They suggested that both miRNAs may be involved in a crosstalk process in the microenvironment, to retain tumor oncogenesis and tumorigenesis, being delivered to gastric juice [102]. In a similar cohort (34 healthy patients, 62 GC patients, 32 atrophic gastritis, 36 gastric ulcer, and 40 superficial gastritis patients), Shao et al. identified that miR-133a was able to distinguish GC patients among healthy individuals with an AUC of 0.907, sensitivity of 85.9%, and specificity of 84.8% [103].

#### 6.2.2. Other Noncoding RNAs

Regarding other noncoding RNAs, some circRNAs [104] and PIWI-interaction RNAs (piRNAs) have also been in the spotlight because of their difficulty to be degraded and their stability. Focusing on piRNAs, in a recent study, Zhou et al. evaluated the role of piR-1245 as an indicator for GC. They obtained and AUC of 0.885 with a sensitivity value of 90.9%, obtaining better values than the traditional indicators for GC (CEA and CA724) [105].

In a similar context, some lncRNAs have also been suggested as biomarkers for GC. Among them, AA17084 stands out. Shao et al. studied a cohort of 45 healthy patients, 39 GC patients, 30 gastric ulcer patients, and 16 chronic atrophic gastritis patients. AA17084 could distinguish GC patients from healthy, gastric ulcer, and chronic atrophic gastritis patients with an AUC of 0.848. In this study, the authors also found an increase in the AA174084 level in the early GC group with a positive detection rate of 57.1% [106]. However, its sensitivity value is still low for its use in daily clinical diagnosis, and combination of other biomarkers could potentially improve the detection rate. A similar situation occurred with other lncRNAs such as RMRP [107] or ABHD11-AS1 [108] that obtained sensitivity values under 57%.

### 6.3. Proteins

Regarding gastric juice proteins, as previously seen in stool samples, GP87 was studied by Qiao et al. in a cohort of 80 healthy patients, 82 GC patients, 20 chronic superficial gastritis patients, 14 patients with ulcer, 32 chronic atrophic gastritis patients, and three patients with polyps. They observed that the positive rate of GP87 in gastric juice was 86.3% for GC and 60% for chronic atrophic gastritis patients, while the rates for the chronic superficial gastritis group and for the normal group were 26.1% (*p* < 0.05) and 16% (*p* < 0.05), respectively [87].

In summary, gastric juice is present only in the stomach. This gives gastric juice biomarkers some obvious advantages for the diagnosis of GC. However, although upper endoscopy is an invasive technique, it is a less invasive strategy than biopsy, which can bring out some interesting biomarkers for GC screening (Table 5).

**Figure 1:**
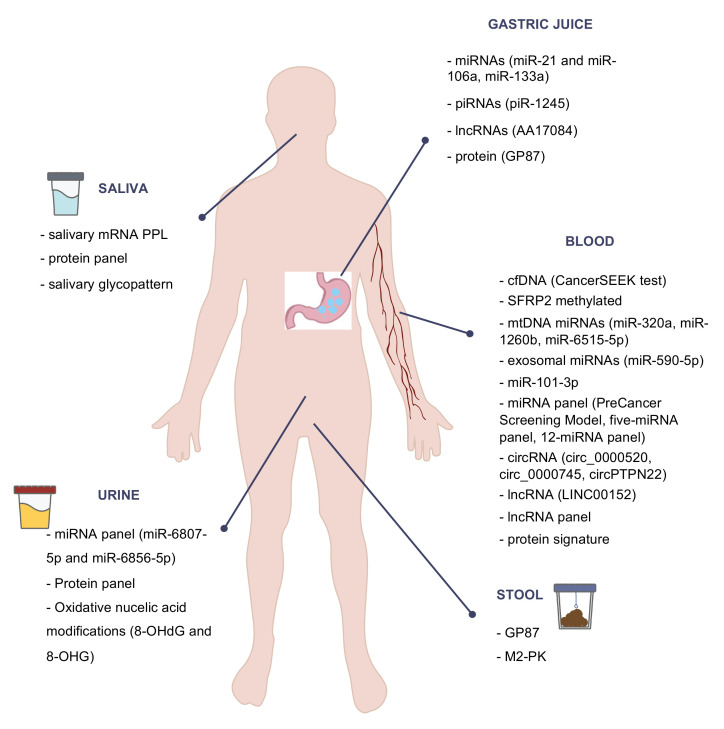
Scheme of some of the most important noninvasive biomarkers for gastric cancer diagnosis and screening.

Figure 1 highlights some of the most important noninvasive biomarkers for GC diagnosis and screening.

## 7. Conclusions

In this review, we described some of the most promising biomarkers that could play a role in early GC diagnosis and screening. Blood biomarkers seem to be one of the most promising, probably because of its acceptance in terms of sampling by general population. On the other hand, urine, stool, or saliva samples need to be further studied, since their easy obtention could improve GC screening, especially saliva. Although gastric juice is obtained invasively, its direct relationship with the stomach makes it quite interesting; hence, further studies focusing on its collection could be useful.

Although there are some interesting biomarkers, the best strategy may be the combination of different biomarkers and even clinical and biochemical parameters to increase the diagnostic accuracy. In this context, miRNA panels can be mentioned as one of the most feasible strategies for the future. However, although some results are very promising, further studies are still needed with a larger sample size, with a greater number of healthy patients representing the real population of screening, and without leaving aside low-prevalence populations, as well as premalignant conditions such as atrophic gastritis, intestinal metaplasia, and dysplasia.

To sum up, the authors predict that, in the near future, a panel of biomarkers obtained in the least invasive way possible will have enough power to diagnose GC and its premalignant lesions in the earliest stages, improving patient survival and quality of life.

## Figures and Tables

**Table 1:** Summary of potential biomarkers for gastric cancer screening and early diagnosis in blood.

Sample	Type	Biomarker	Patients	AUC (95% CI)	Sensitivity (%)	Specificity (%)	Ref.
**Blood Biomarkers**							
Blood (serum)	DNA	Circulating free DNA	216 patients (92 healthy patients and 124 primary gastric cancer patients)	0.94 (0.91–0.97)	78.96	91.81	[15]
Blood (plasma)	DNA	Circulating free DNA	132 patients (34 healthy patients, 34 GC patients, and 64 benign gastric patients)	0.99	96.67	94.11	[16]
Blood (plasma)	DNA + protein	Mutations in circulating free DNA (16 genes + 8 proteins)	1817 patients (812 healthy patients, 68 GC patients, 209 breast cancer patients, 388 colorectal cancer patients, 45 esophagus cancer patients, 44 liver cancer patients, 104 lung cancer patients, 54 ovary cancer patients, and 93 pancreatic cancer)	0.91 (0.90–0.92)	70.00 for GC	99.00	[18]
Blood (plasma)	DNA	Methylated RUNX3	111 patients (61 healthy patients and 50 GC patients)	0.69	50.00	80.30	[20]
Blood (plasma)	DNA	Methylated SFRP2	236 patients (50 healthy patients, 92 GC patients, 16 intestinal metaplasia patients, 26 gastric fundic gland polyp patients, 13 small adenoma patients, and 39 hyperplastic polyp patients)	0.78 (0.71–0.86)	60.90	86.0	[21]
Blood (plasma)	DNA	Methylated RPRML	50 patients (25 healthy patients and 25 GC patients)	0.73 (0.58–0.87)	56.00	88.00	[22]
Blood	DNA	mtDNA	276 patients (48 healthy patients, 74 patients with non-atrophic gastritis, 31 patients with intestinal metaplasia, and 123 GC patients)	-	47.00	80.00	[24]
Blood (plasma)	miRNA	hsa-miR-320a	3408 patients (3174 healthy patients and 234 GC patients)	0.96–1.00	99.10–100.00	88.8–100.00	[26]
Blood (plasma)	miRNA	hsa-miR-1260b	3408 patients (3174 healthy patients and 234 GC patients)	0.97–1.00	97.40–98.00	89.60–100.00	[26]
Blood (plasma)	miRNA	hsa-miR-6515-5p	3408 patients (3174 healthy patients and 234 GC patients)	0.95–0.99	92.20–98.00	88.70–92.70	[26]
Blood (serum)	miRNA	Exosomal miR-590-5p	218 patients (50 healthy patients and 168 GC patients)	0.81	63.70	86.00	[29]
Blood (plasma)	miRNA	Exosomal miR-1246	165 patients (50 healthy patients, 30 patients with benign gastric disease, and 85 GC patients)	0.91 (0.86–0.96)	82.30	86.00	[30]
Blood (serum)	miRNA	miR-101-3p	197 patients (50 healthy patients, 86 GC patients and 61 atrophic gastritis patients)	0.88	72.09	86.49	[33]
Blood (plasma)	miRNA	miR-376c	173 patients (108 healthy patients and 65 GC patients)	0.77	71.00	78.00	[31]
Blood (plasma)	miRNA	miR-21	100 patients (50 healthy patients and 50 GC patients)	0.91 (0.87–0.97)	88.40	79.60	[32]
Blood (plasma)	miRNA panel	Pre-Cancer Screening Model (age, miR-19a-3p and miR-483-5p)	180 patients (70 gastritis subjects, 20 low-grade intraepithelial neoplasia, 34 high-grade intraepithelial neoplasia, and 56 early GC)	Early GC and prec. lesions: 0.84 (0.77–0.90)	Early GC and prec. lesions: 87.70	Early GC and prec. lesions: 62.80	[34]
Blood (plasma)	miRNA panel	miR-16, miR-25, miR-92a, miR-451 and miR-486-5p	320 patients (160 healthy patients, 124 non cardia GC patients, and 36 cardia GC patients)	Non-cardia GC: 0.81	Non-cardia GC: 72.90	Non-cardia GC: 89.20	[35]
Blood (serum)	miRNA panel	12 miRNA panel	5248 patients (4803 healthy patients and 445 GC)	0.85 (0.81–0.88)	87.00	68.40	[36]
Blood (serum)	miRNA panel + other factors	miRNA panel + age + *H. pylori* serology + pepsinogen I/II ratio	5248 patients (4803 healthy patients and 445 GC)	0.88	87.00	69.40	[36]
Blood (plasma)	circRNA	hsa_circ_0000520	62 patients (17 healthy patients and 45 GC)	0.90	82.35	84.44	[37]
Blood (plasma)	circRNA	Exosomal circ_0065149	80 patients (41 healthy patients and 39 early GC)	0.64	48.70	90.20	[38]
Blood (plasma)	circRNA	circ_0000745	120 patients (60 healthy patients and 60 GC patients)	0.68	85.50	45.00	[39]
Blood (plasma)	circRNA	circ_0000745 + CEA	120 patients (60 healthy patients and 60 GC patients)	0.78	80.00	63.30	[39]
Blood (plasma)	circRNA	circPTPN22	294 patients (104 healthy patients, 120 plasma of GC patients, and 70 gastritis patients)	0.857 (0.808–0.907)	78.00	84.00	[40]
Blood (plasma)	circRNA + antigens	circPTPN22 + CEA + CA199	294 patients (104 healthy patients, 120 plasma of GC patients, and 70 gastritis patients)	0.89	83.00	87.00	[40]
Blood (serum)	lncRNA	multi-lncRNA diagnostic panel (CUDR, LSINCT-5 and PTENP1)	231 patients (124 healthy patients, 110 GC patients, 9 early-stage GC, 21 later-stage GC, and 15 gastric peptic ulcer patients without GC)	GC:0.83 Early-GC: 0.83 Later-GC: 0.83	GC: 81.80 Early-GC: 77.80 Later-GC: 85.70	GC: 85.20 Early-stage: 97.00 Later-stage: 84.80	[41]
Blood (plasma)	lncRNA	TINCR, CCAT2, AOC4P, BANCR and LINC00857	321 patients (110 healthy patients, 162 GC patients, 28 prec. Patients, and 21 GIST)	GC: 0.90 (0.86–0.95) Prec: 0.82 (0.71–0.92) GIST: 0.80 (0.68–0.91	GC: 0.81 (0.71–0.89) Prec: 0.68 (0.50–0.82) GIST: 0.68 (0.50–0.82)	GC: 0.86 (0.77–0.93) Prec: 0.89 (0.72–0.98) GIST: 0.86 (0.64–0.97)	[42]
Blood (plasma)	lncRNA	Exosomal LINC00152	191 patients (81 healthy patients, 79 GC patients, and 31 gastric epithelial dysplasia patients)	0.66 (0.57–0.74)	48.10	85.20	[43]
Blood (plasma)	lncRNA	Exosomal lncUEGC1	144 patients (65 healthy patients, 18 CAG, and 61 stage I/II GC patients)	0.88	-	-	[44]
Blood (serum)	lncRNA	Exosomal lncRNA HOTTIP	246 patients (120 healthy patients and 126 GC patients)	0.83	69.80	85.00	[45]
Blood (serum)	lncRNA	Exosomal lncRNA HOTTIP + CEA + CA19-19 + CA72-4	246 patients (120 healthy patients and 126 GC patients)	0.87	-	-	[45]
Blood (plasma)	Protein	Protein signature (ATP5B-ATP5O-NDUFB4-NDUFB8)	71 patients (34 healthy patients and 37 GC patients)	0.79	-	-	[47]
Blood (serum)	Protein panel	Protein signature (VEGF, ADAM8, IgG to *H. pylori* status, serum pepsinogen I, and pepsinogen II)	523 patients (238 healthy patients and 285 GC patients)	0.85 (0.77–0.93)	88.60	83.20	[48]
Blood (serum)	Protein	SLC6A3	220 patients (56 healthy patients, 113 GC patients, and 51 patients with polyps)	0.72 (0.64–0.80)	55.75	78.57	[49]
Blood (serum)	Protein panel	SLC6A3, CEA and CA19-9	220 patients (56 healthy patients, 113 GC patients, and 51 patients with polyps)	0.82 (0.75–0.88)	84.07	58.93	[49]
Blood (serum)	Protein	anti-GRP78 autoantibody	866 patients (433 healthy patients and 433 GC patients)	0.67	35.30	-	[46]
Blood (plasma)	Protein	Q8NBP7	30 patients (15 healthy controls and 15 early GC)	0.702	60.0	80.0	[50]
Blood (plasma)	Protein	P00441	30 patients (15 healthy controls and 15 early GC)	0.707	93.3	46.7	[50]
Blood (plasma)	Protein	Q86UD1	30 patients (15 healthy controls and 15 early GC)	0.711	46.7	100	[50]
Blood (plasma)	Protein	A0A2R8Y7X9	30 patients (15 healthy controls and 15 early GC)	0.796	80.0	86.7	[50]
Blood (plasma)	Protein	P62979	30 patients (15 healthy controls and 15 early GC)	0.724	86.7	66.7	[50]
Blood (plasma)	Protein	A0A0G2JMC9	30 patients (15 healthy controls and 15 early GC)	0.747	60.0	93.3	[50]
Blood (plasma)	Protein	P08493	30 patients (15 healthy controls and 15 early GC)	0.813	73.3	80.0	[50]
Blood (plasma)	Protein	P16157	30 patients (15 healthy controls and 15 early GC)	0.653	46.7	86.7	[50]
Blood (plasma)	Protein	A0A087WTY6	30 patients (15 healthy controls and 15 early GC)	0.64	80.0	53.3	[50]
Blood (plasma)	Protein	P14207	30 patients (15 healthy controls and 15 early GC)	0.689	60.0	80.0	[50]
Blood (plasma)	Protein	Q9H939	30 patients (15 healthy controls and 15 early GC)	0.573	73.3	53.3	[50]
Blood (plasma)	Protein panel	11 protein panel	30 patients (15 healthy controls and 15 early GC)	0.711	66.7	86.7	[50]
Blood (serum)	Protein	PGI	72 patients (35 healthy controls, 34 CAG and 3 GC)	0.659	66.7	85.3	[51]
Blood (serum)	Protein	PGI:II ratio	72 patients (35 healthy controls, 34 CAG and 3 GC)	0.902	83.3	77.9	[51]
Blood (serum)	Protein	TFF3	567 patients (325 healthy controls and 242 GC patients)	0.890	80.9	81.0	[53]
Blood (plasma)	Protein	TFF3	86 patients (44 healthy controls and 42 stage I GC patients)	0.703	83.3	54.5	[54]
Blood (serum)	Protein	Hs-CRP + PGI:II ratio	378 patients (69 healthy controls, 47 CAG, 75 IM, 41 early GC, 43, AGC, and 87 metastasis GC)	-	AGC: 67.0 IM: 67.0	AGC: 85.0 IM: 72.0	[57]
Blood (serum)	Protein	Hs-CRP	378 patients (69 healthy controls, 47 CAG, 75 IM, 41 early GC, 43, AGC, and 87 metastasis GC)	-	AGC: 67.0	AGC: 85.0	[57]

GC, gastric cancer; CAG, chronic atrophic gastritis; IM, intestinal metaplasia; AGC, locally advanced gastric cancer; prec, precancerous; GIST, gastrointestinal stromal tumor; AUC, area under the curve; Ref, reference; -, no data available.

**Table 2:** Summary of potential saliva biomarkers for gastric cancer screening and early diagnosis.

Sample	Type	Biomarker	Patients	AUC (95% CI)	Sensitivity (%)	Specificity (%)	Ref.
**Saliva Biomarkers**							
Saliva	miRNA + mRNA	3 mRNAs (SPINK7, PPL, and SEMA4B) and 2 miRNAs (MIR140-5p and MIR301a) + demographic	294 patients (131 healthy patients and 163 GC patients)	0.87 (0.80–0.93)	82.00	77.00	[69]
Saliva + blood	Protein + mRNA	CEA + mRNA PPL	400 patients (200 healthy patients and 200 GC patients)	-	92.00	87.00	[70]
Saliva	Protein	CSTB + TPI1 + DMBT1	80 patients (40 healthy patients and 40 GC patients)	0.93	85.00	80.00	[71]
Saliva	Protein glycosylation	Salivary glycopattern (for GC VVA and SBA) (for atrophic gastritis DSA and LEL)	201 patients (60 healthy patients, 87 GC patients, and 54 atrophic gastritis patients)	GC: 0.89 Atrophic gastritis: 0.83	GC: 0.96 Atrophic gastritis: 0.92	GC:0.80 Atrophic gastritis: 0.72	[72]

GC, gastric cancer; AUC, area under the curve; Ref, reference; -, no data available.

**Table 3:** Summary of potential urine biomarkers for gastric cancer screening and early diagnosis.

Sample	Type	Biomarker	Patients	AUC (95% CI)	Sensitivity (%)	Specificity (%)	Ref.
**Urine Biomarkers**							
Urine	miRNA	miR-376c	173 patients (108 healthy patients and 65 GC patients)	0.70	60.00	64.00	[31]
Urine	miRNA panel	miR-6807-5p/miR-6856-5p/*H. pylori* status	550 patients (302 healthy patients and 248 GC patients)	GC: 0.89 (0.82–0.95) Stage I GC: 0.75 (0.68–0.81)	GC: 76.90 Stage I GC: -	GC: 88.90 Stage I GC: -	[78]
Urine	Protein	MMP-9/NGAL complex and ADAM12	70 patients (35 healthy patients and GC patients)	0.83 (0.72–0.93)	-	-	[79]
Urine	Protein panel	uTFF1/uADAM12/*H. pylori* status for males; uTFF1/uBARD1/*H. pylori* status for females	282 patients (138 healthy patients 144 GC patients)	Male: 0.86 (0.81–0.91) Female: 0.89 (0.80–0.99)	-	-	[80]
Urine	DNA	8-OHdG	130 patients (70 healthy patients and 60 GC patients)	0.78 (0.70–0.86)	-	-	[81]
Urine	DNA	8-OHG	130 patients (70 healthy patients and 60 GC patients)	0.84 (0.77–0.91)	-	-	[81]

GC, gastric cancer; AUC, area under the curve; Ref, reference; -, no data available.

**Table 4:** Summary of potential fecal biomarkers for gastric cancer screening and early diagnosis.

Sample	Type	Biomarker	Patients	AUC (95% CI)	Sensitivity (%)	Specificity (%)	Ref.
**Stool Biomarkers**							
Stool	Protein	GP87	231 patients (80 healthy patients, 82 GC patients, 20 chronic superficial gastritis patients, 14 patients with ulcer, 32 CAG patients, and 3 patients with polyps)	-	GC: 79.30 CAG: 84.40	-	[87]
Stool	Protein	M2-PK	74 patients (20 healthy patients and 54 GC patients)		50.47	-	[88]

GC, gastric cancer; CAG, chronic atrophic gastritis; AUC, area under the curve; Ref, reference; -, no data available.

**Table 5:** Summary of potential biomarkers in gastric juice for gastric cancer screening and early diagnosis.

Sample	Type	Biomarker	Patients	AUC (95% CI)	Sensitivity (%)	Specificity (%)	Ref.
**Gastric Juice Biomarkers**							
Gastric juice	DNA	*BARHL2*	30 patients (10 healthy patients, 10 early GC, and 10 advanced GC)	0.92	90.0	100.0	[99]
Gastric juice	miRNA	miR-129-1-3p and miR-129-2-3p	141 patients (47 healthy patients, 42 GC patients, 34 gastric ulcer patients, and 18 atrophic gastritis patients)	0.66	68.70	71.90	[100]
Gastric juice	miRNA	miR-421	141 patients (47 healthy patients, 42 GC patients, 34 gastric ulcer patients, and 18 atrophic gastritis patients)	0.77 (0.68–0.85)	71.40	71.70	[101]
Gastric juice	miRNA	miR-21	141 patients (47 healthy patients, 42 GC patients, 34 gastric ulcer patients, and 18 atrophic gastritis patients)	0.97	85.70	97.80	[102]
Gastric juice	miRNA	miR-106a	141 patients (47 healthy patients, 42 GC patients, 34 gastric ulcer patients, and 18 atrophic gastritis patients)	0.87	73.80	89.30	[102]
Gastric juice	miRNA	miR-133a	204 patients (34 healthy patients, 62 GC patients, 32 atrophic gastritis, 36 gastric ulcer, and 40 superficial gastritis patients)	0.91 (0.86–0.96)	85.90	84.80	[103]
Gastric juice	circRNA	circ_0014717	122 patients (38 healthy patients, 30 gastric ulcer patients, 15 CAG, and 39 GC patients)	-	-	-	[104]
Gastric juice	piRNA	piR-1245	132 patients (66 healthy patients and 66 GC patients)	0.89	90.90	74.20	[105]
Gastric juice	lncRNA	RMRP	130 patients (45 healthy patients, 39 GC patients, 30 gastric ulcer patients, and 16 CAG patients)	0.70	56.40	75.40	[107]
Gastric juice	lncRNA	AA174084	130 patients (45 healthy patients, 39 GC patients, 30 gastric ulcer patients, and 16 CAG patients)	0.85 (0.78–0.92)	46.00	93.00	[106]
Gastric juice	lncRNA	lncRNA-ABHD11-AS1	39 GC patients, 30 patients with gastric ulcers, 16 patients with atrophic gastritis, and 45 patients with normal mucosa/minimal gastritis	0.65 (0.54–0.77)	41.00	93.40	[108]
Gastric juice	Protein	GP87	231 patients (80 healthy patients, 82 GC patients, 20 chronic superficial gastritis patients, 14 patients with ulcer, 32 CAG patients, and 3 patients with polyps)	-	GC: 86.30 CAG: 60.00	-	[87]

GC, gastric cancer; CAG, chronic atrophic gastritis; AUC, area under the curve; Ref, reference; -, no data available.

## Data Availability

Not applicable.
